# Spectroscopic Study of Terahertz Generation in Mid-Infrared Quantum Cascade Lasers

**DOI:** 10.1038/srep21169

**Published:** 2016-02-16

**Authors:** Yifan Jiang, Karun Vijayraghavan, Seungyong Jung, Aiting Jiang, Jae Hyun Kim, Frederic Demmerle, Gerhard Boehm, Markus C. Amann, Mikhail A. Belkin

**Affiliations:** 1 Department of Electrical and Computer Engineering, The University of Texas at Austin, Microelectronics Research Center, 10100 Burnet Road, Austin, TX 78758, U.S.A; 2 Walter Schottky Institut, Technische Universität München, Am Coulombwall 4, 85748, Garching, Germany

## Abstract

Terahertz quantum cascade laser sources based on intra-cavity difference-frequency generation are currently the only room-temperature mass-producible diode-laser-like emitters of coherent 1–6 THz radiation. Device performance has improved dramatically over the past few years to reach milliwatt-level power output and broad tuning from 1.2 to 5.9 THz, all at room-temperature. Terahertz output in these sources originates from intersubband optical nonlinearity in the laser active region. Here we report the first comprehensive spectroscopic study of the optical nonlinearity and investigate its dependence on the mid-infrared pump frequencies. Our work shows that the terahertz generation efficiency can vary by a factor of 2 or greater depending on the spectral position of the mid-infrared pumps for a fixed THz difference-frequency. We have also measured for the first time the linewidth for transitions between the lower quantum cascade laser states, which is critical for determining terahertz nonlinearity and predicting optical loss in quantum cascade laser waveguides.

The terahertz (THz) spectral range (0.3–10 THz) has a proven potential for a variety of applications, such as spectroscopy, communications, and security screening^1^,^2^,^3^,^4^. Room-temperature THz sources analogous to diode lasers in the near-infrared/visible or quantum cascade lasers (QCL) in the mid-infrared (mid-IR), i.e. electrically-pumped, compact, widely-tunable, and suitable for low-cost production, are highly desired for feasible and inexpensive THz systems. Despite significant development of THz QCLs over the past 12 years^5^,^6^,^7^,^8^, their operating temperatures are limited to 200 K for pulsed operation^9^ and 130 K for continuous-wave (CW) operation^10^.

THz sources based on intra-cavity difference-frequency generation (DFG) in dual-wavelength mid-IR QCLs^11^,^12^,^13^,^14^,^15^,^16^,^17^,^18^,^19^,^20^ are currently the only electrically-pumped mass-producible monolithic semiconductor laser sources that can operate at room temperature in 1–6 THz region. With the introduction of waveguides designed for Cherenkov phase-matching^12^,^13^, these devices, referred to as THz DFG-QCLs, have achieved dramatic improvement in power and tuning bandwidth. A single ridge-waveguide THz DFG-QCL can now output nearly 2 mW of THz peak power in pulsed mode and microwatt-level THz power in CW mode, all at room temperature^14^,^15^. When integrated in an external cavity setup, these devices can provide widely-tunable narrowband THz emission from 1 to 6 THz at room temperature^13^,^16^. A variety of monolithic electronically-tunable THz DFG-QCL sources were also demonstrated at room temperature^17^,^18^,^19^.

Terahertz generation in THz DFG-QCLs is produced via the nonlinear mixing of the two mid-IR pump fields generated in the QCL active region. The active region is also designed to have resonant optical nonlinearity *χ*^(2)^ associated with transitions between the upper laser states and the manifold of the lower laser states as shown in Fig. 1(a,b)^11^,^12^,^13^,^14^,^15^,^16^,^17^,^18^,^19^,^20^,^21^,^20^. Terahertz power output (W_THz_) scales with the product of mid-IR pump powers (W_1_ and W_2_) and the square of the magnitude of *χ*^(2)^ 11,12,13,14,15,16,17,18,19,20:


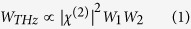


The ability to accurately predict and maximize *χ*^(2)^ in the active region is critical for further advancement of THz DFG-QCL technology.

Referring to the energy state labelling shown in Fig. 1 and keeping only resonant terms, the quantum mechanical expression for *χ*^(2)^ for THz DFG is given as^20^.


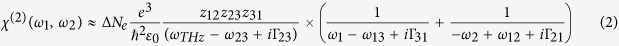


where *ω*_1_ and *ω*_2_ are the frequencies of mid-IR pumps, *ω*_THz_ = *ω*_1_ − *ω*_2_ is THz difference-frequency, Δ*N*_*e*_ is the population inversion density, 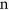 and 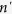 are the lower state levels (levels 2,3, and other lower laser levels shown in Fig. 1), ez_ij_, *ω*_ij_, and Γ_ij_ are the transition dipole moments, transition frequency, and transition linewidth factor between states 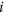 and 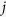. Equation (2) shows that *χ*^(2)^ has a strong 2-dimensional (2D) dependence on the mid-IR pump frequencies. However, no work has been performed so far to experimentally measure the dependence of *χ*^(2)^ on *ω*_1_ and *ω*_2_ in THz DFG-QCLs. The linewidth factor Γ_23_ for THz transitions between the lower laser states has also not been experimentally quantified. Determining the linewidth factor Γ_23_ is critical for improving the modeling accuracy of *χ*^(2)^ and optical loss in the lower laser levels structure of QCLs. We note that it is nearly impossible to measure Γ_23_ using linear absorption techniques because both states 2 and 3 are designed to be quickly depopulated under QCL operating bias.

Here we report the results of the first 2D nonlinear spectroscopic study of THz generation in DFG-QCLs. We obtain the values for linewidth Γ_23_ and experimentally show that the THz generation efficiency at a fixed THz frequency (*ω*_THz_ = *ω*_1_ − *ω*_2_) for a specific applied bias field can vary by a factor of 2 or more, depending on the choice of *ω*_1_ and *ω*_2_. Our results and the 2D spectroscopic technique presented here are expected to play a crucial role in the analysis and future optimization of the performance of the only room-temperature semiconductor-laser-like sources of THz radiation.

## Results

The experimental setup used in this work is presented in Fig. 2(a) and additional details are given in Methods. We performed independent tuning of the two mid-IR pumps using a dual-diffraction-grating external cavity (EC) setup. The positions of the diffraction gratings were computer controlled. Our setup may be viewed as an extension of Littrow-type external cavity setup used for mid-IR QCLs^21^,^22^. It is also similar to a dual-grating EC setup used previously for dual-color tuning of near-infrared diode lasers^23^. Cherenkov THz DFG emission from the device was collected through a 30-degree polished device substrate facet as shown schematically in Fig. 2(a) 12,15. Terahertz power measurements were performed in a N_2_-purged environment with a calibrated liquid-helium cooled bolometer. Individual mid-IR pump powers were spectrally separated using interference filters and measured with a calibrated thermopile detector. All measurements were performed at room temperature using 50 ns current pulses at a 50 kHz repetition frequency. Further details of the experimental setup are provided in Methods.

**Figure 1:**
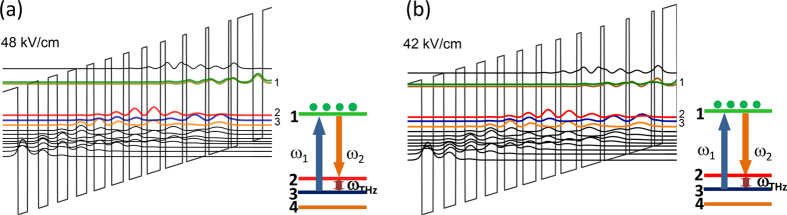
Active region band structure of two-stack active region design under applied bias computed using a Schrödinger solver^26^. (**a**) Conduction band diagram of one period of λ = 8.5 μm active region at 48 kV cm^−1^ bias field, close to the rollover point. Inset: schematic diagram showing the DFG process between the electron states in this structure. Transition dipole matrix elements and energy spacing between states 1, 2, and 3 are computed to be z_12_ = 2.2 nm, z_13_ = 1.9 nm, z_23_ = 8.0 nm, E_12_ = 135 meV, E_13_ = 154 meV. (**b**) Conduction band diagram of one period of λ = 9.5 μm active region at 42 kV cm^−1^ bias field, close to the rollover point. Inset: schematic diagram showing the DFG process between the electron states in this structure. Transition dipole matrix elements and energy spacing between states 1, 2, and 3 are computed to be: z_12_ = 2.3 nm, z_13_ = 2.2 nm, z_23_ = 9.0 nm, E_12_ = 121 meV, E_13_ = 137 meV.

Cherenkov THz DFG-QCL chips used in our work were identical to devices reported in ref. 13. The laser structure was grown by molecular beam epitaxy on a 350-μm-thick semi-insulating InP substrate. The laser active region was comprised of 33 repetitions of the bandstructure shown in Fig. 1(a) designed to have a gain peak at λ = 8.5 μm followed by 33 repetitions of the bandstructure shown in Fig. 1(b) that was designed to have a gain peak at λ = 9.5 μm. This dual-stack active region is designed to provide a broad gain bandwidth for mid-IR pump tuning with a difference-frequency as high as ~6 THz^13^,^16^. Devices were fabricated into 14 to 32 μm wide ridge-waveguide lasers using a dry etching process. A lateral current extraction scheme was used due to the non-conductive nature of the semi-insulating InP substrate^12^,^13^. Further details of the device structure are provided in Methods. The results discussed below are all obtained with a 22-μm-wide, 1.7-mm-long device; however, similar results were obtained with devices of other dimensions.

**Figure 2:**
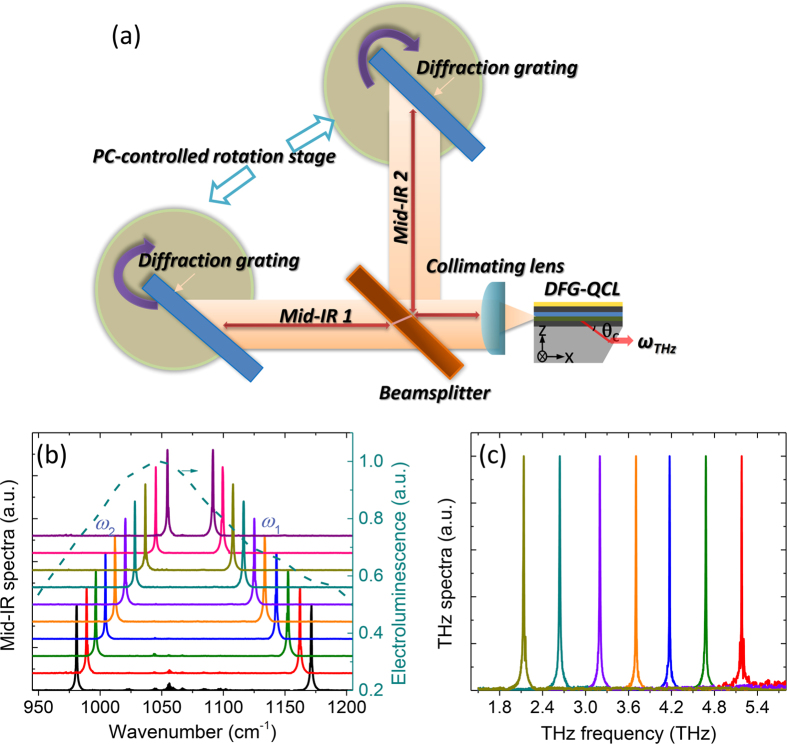
Details of the laser tuning setup. (**a**) Schematic of the dual-grating Littrow-type external cavity used in this work. The back facets of the laser chips are coated with a two-layer anti-reflection coating (AR) as described in ref. 16. The collimating lens is an AR-coated aspheric lens with a focal length of 1.87 mm and a numerical aperture of 0.85. The non-polarizing beam splitter is made of ZnSe and transmits (reflects) approximately 50% of the incident light. Two gold-coated 150 grooves per mm diffraction gratings are used to provide frequency-tunable feedback for the two mid-IR pump frequencies (*ω*_1_ and *ω*_2_) independently. (**b**) Mid-IR emission spectra of a THz DFG-QCL in the setup depicted in (**a**) for several different grating positions. In this example one diffraction grating tunes mid-IR pump frequency *ω*_2_ from 980 cm^−1^ to 1054 cm^−1^ and the other diffraction grating tunes mid-IR pump frequency *ω*_1_ from 1091 cm^−1^ to 1170 cm^−1^. The electroluminescence spectrum of the laser material is shown with a dashed line. (**c**) Selected THz emission spectra of the device recorded for the same diffraction grating positions as in (**b**).

**Figure 3:**
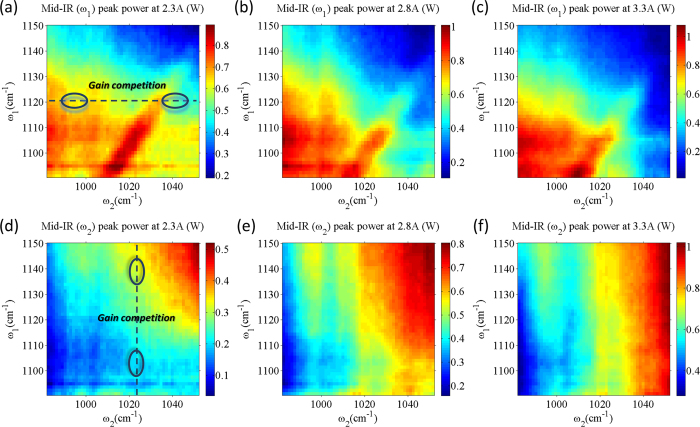
Mid-IR peak power output (in watts) as a function of mid-IR pump frequencies *ω*_1_ and *ω*_2_. The pump frequencies *ω*_1_ and *ω*_2_ are selected by the angle of two external diffraction gratings. Panels (**a–c**) show the peak power output of *ω*_1_ pump at pump currents of 2.3 A, 2.8 A and 3.3 A, respectively. Panels (**d–f**) show the peak power output of *ω*_2_ pump at pump currents of 2.3 A, 2.8 A and 3.3 A, respectively.

Selected mid-IR tuning spectra from the THz DFG-QCL in the EC setup are shown in Fig. 2(b) for different grating positions. The corresponding THz emission spectra of the device are shown in Fig. 2(c). For the 2D spectroscopy results presented in Fig. 3, the frequency of mid-IR pump 1 (*ω*_1_) was varied between 1091 cm^−1^ to 1151 cm^−1^ and the frequency of pump 2 (*ω*_2_) was varied, independently, between 981 cm^−1^ to 1052 cm^−1^. THz DFG frequency corresponds to the frequency separation between the two mid-IR pumps (*ω*_THz_ = *ω*_*1*_ − *ω*_*2*_) and could be tuned from 1.17 THz (39 cm^−1^) to 5.10 THz (170 cm^−1^).

The peak power of mid-IR pump 1 and pump 2 as a function of pump frequencies selected by external gratings is shown in Fig. 3(a–f), respectively. Measurements were performed at three different currents of 2.3 A, 2.8 A, and 3.3 A (current density of 6.2 kA/cm^2^, 7.5 kA/cm^2^, and 8.8 kA/cm^2^), corresponding to near laser threshold, mid dynamic range, and near laser rollover, respectively. The effect of gain competition between the two mid-IR pumps can be observed: in Fig. 3(a), the intensity of pump 1 decreases as the frequency of pump 2 is tuned towards *ω*_1_, and in Fig. 3(d), the intensity of pump 2 decreases as the frequency of pump 1 is tuned towards *ω*_2_. Similar gain competition effects are seen at higher bias currents in Fig. 3(b,c,e,f). Gain competition is an intrinsic effect in all dual-color semiconductor lasers and has been reported previously for THz DFG-QCLs^16^,^24^,^25^. It can be seen in Fig. 3(a,d) that dual-color mid-IR lasing is possible for a wide range of *ω*_1_ and *ω*_2_ frequencies despite gain competition. The balance of mid-IR pump powers can be managed by controlling the individual round-trip gain and/or loss for the two pumps^19^. We further note that even with gain competition, both mid-IR pumps operate in the TM_00_ mode, as confirmed by far field measurements, due to spatial filtering of EC feedback at device aperture.

**Figure 4:**
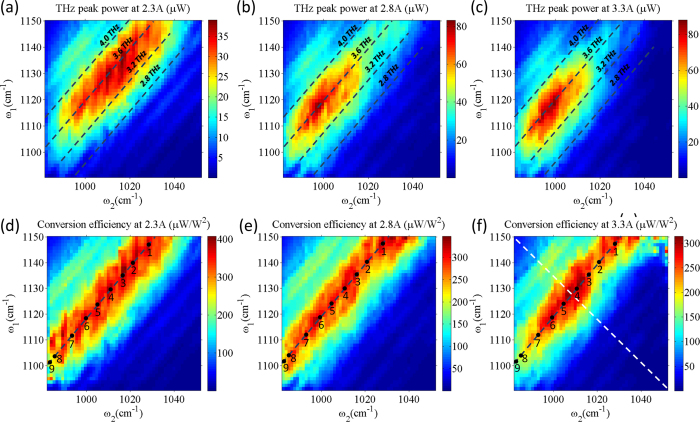
THz DFG performance of the device. Panels (**a–c**) show the THz peak power output (in μW) of the device at pump currents of 2.3 A, 2.8 A and 3.3 A, respectively, as a function of mid-IR pump frequencies *ω*_1_ and *ω*_2_. Panels (**d–f**) show mid-IR-to-THz conversion efficiency (in μW/W^2^) of the device at pump currents of 2.3 A, 2.8 A and 3.3 A, respectively, as a function of mid-IR pump frequencies *ω*_1_ and *ω*_2_. Black dashed lines in all panels correspond to the sets of data points with the constant values of THz DFG frequency at 2.8, 3.2, 3.6 and 4.0 THz. Points labelled with numbers indicate frequencies of the two mid-IR pumps for which we performed more detailed measurements of THz power output as a function of mid-IR pump powers as shown in Fig. 5. White dashed line in (**f**) indicates the set of data points used to determine Γ_23_ linewidth as shown in Fig. 6.

The dependence of THz peak power on the two mid-IR pump frequencies is shown in Fig. 4(a–c). Similar to data in Fig. 3(a–f), measurements for the data in Fig. 4(a–c) were performed at three different pump currents of 2.3 A, 2.8 A, and 3.3 A (current density of 6.2 kA/cm^2^, 7.5 kA/cm^2^ and 8.8 kA/cm^2^). Figure 4(d–f) shows the mid-IR-to-THz conversion efficiency *η*(*ω*_1_, *ω*_2_) for the three different bias currents, defined as the ratio of THz power output from a device to the product of mid-IR pump powers,


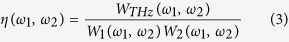


where W_1_(*ω*_1_, *ω*_2_) is the power of mid-IR pump 1 shown in Fig. 3(a–c), W_2_(*ω*_1_, *ω*_2_) is the power of mid-IR pump 2 shown in Fig. 3(d–f), and W_THz_(*ω*_1_, *ω*_2_) is the THz output power shown in Fig. 4(a–c). The dashed lines in Fig. 4(a–c) represent positions of the same THz difference frequency of 2.8 THz, 3.2 THz, 3.6 THz and 4.0 THz. It can be seen in Fig. 4(d–f) that the mid-IR-to-THz conversion efficiency profile as a function of *ω*_1_ and *ω*_2_ does not stay constant and the maximum mid-IR-to-THz conversion efficiency is achieved for different combinations of mid-IR pump frequencies as the device bias changes. This is true even if the THz difference-frequency stays the same.

**Figure 5:**
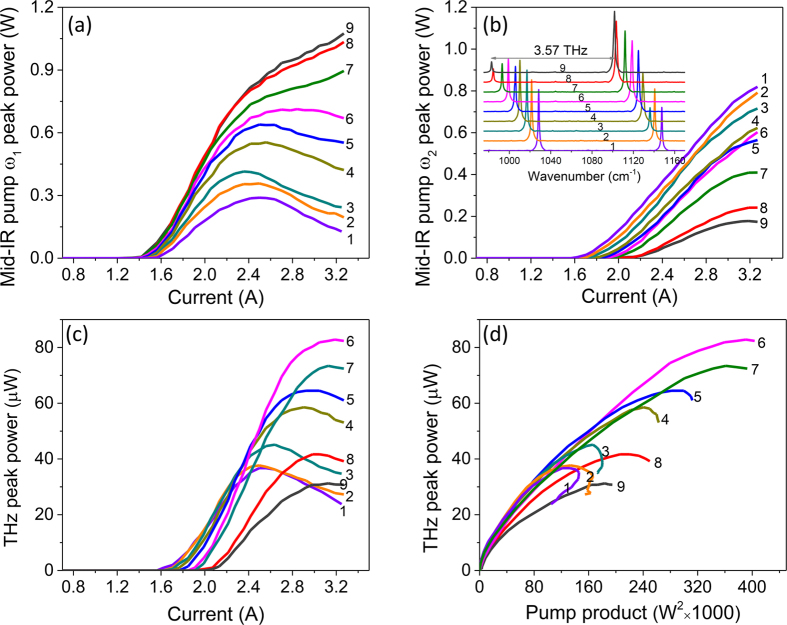
Device performance at a fixed difference-frequency of 3.57 THz with 9 different combinations of *ω*_1_ and *ω*_2_ (indicated with 9 black dots in Fig. 4(d–f)). Panels (**a,b**) show the power of mid-IR pump *ω*_1_ and pump *ω*_2_, respectively, as a function of current through the device. Inset in (**b**) shows the mid-IR emission spectra at pump current of 3.3 A. Panel (**c**) shows THz light-output-current characteristics of the device and (**d**) shows THz power output as a function of the product of the mid-IR pump powers, for 9 different combinations of *ω*_1_ and *ω*_2_.

To illustrate this point further, we performed mid-IR and THz light-output-current characterization of the device at 9 different combinations of *ω*_1_ − *ω*_2_ that fixed the THz difference-frequency at 3.57 THz. These *ω*_1_ and *ω*_2_ combinations are indicated with black dots in Fig. 4(d–f). Figure 5(a,b) show the power of mid-IR pump 1 and pump 2 as a function of current through the device for the 9 different combinations of *ω*_1_ − *ω*_2_; the emission spectra at a pump current of 3.3 A for the same 9 combinations of *ω*_1_ − *ω*_2_ are shown in the inset of Fig. 5(b). Figure 5(c) shows the THz light-output-current characteristics of the device producing 3.57 THz difference-frequency and Fig. 5(d) plots THz power output as a function of the product of the two mid-IR pump powers, obtained from the data shown in Fig. 5(a,b), for the same combinations of *ω*_1_ and *ω*_2_. From Eq. (1), a linear dependence between the THz power output and mid-IR pump power product is expected under the assumption of a constant *χ*^(2)^. However, Fig. 5(d) shows that this is not the case experimentally. Moreover, Fig. 5(d) shows that the mid-IR-to-THz conversion efficiency can vary by a factor of 2 or more depending on the choice of mid-IR pump frequencies for fixed 3.57 THz difference-frequency emission. To explain the results, we need to consider the dependence of *χ*^(2)^ on the mid-IR pumps frequencies and the bias voltage. Indeed, Eq. (2) shows that as transition energies between subbands 1, 2 and 3 change with applied bias, the value of *χ*^(2)^ and its dependence on *ω*_1_ and *ω*_2_ may vary significantly.

## Discussion

To provide qualitative explanation of the results in Fig. 5, we computed the bandstructures of the two stacks (shown in Fig. 1) that comprise the active region for different bias voltages: near the threshold alignment point, in the middle of the dynamic range, and near the rollover point (specifically, at 41 kV cm^−1^, 44 kV cm^−1^, and 47 kV cm^−1^, respectively, for stack 1 and 46 kV cm^−1^, 49 kV cm^−1^, and 52 kV cm^−1^, respectively, for stack 2). Transition energies and transition dipole moments between states 1, 2, and 3 were then computed from the bandstructures^26^ and Eq. (2) was used to calculate *χ*^(2)^(*ω*_1_, *ω*_2_) in each of the active region stack. In this calculation, the transition linewidth between upper and lower laser states Γ_21_ and Γ_31_ were assumed to be 15 meV for both stacks based on electroluminescence measurements^13^, while the linewidth Γ_23_ for the transition between lower laser states was varied from 2 meV to 8 meV. We have also assumed a constant Δ*N*_*e*_ = 8.5 × 10^14^ cm^−3^ in accordance to the value used for similar devices in ref. 13. We note that the data in Fig. 5(a,b) shows that threshold current density (and, correspondingly, Δ*N*_*e*_) in our device may change by up to ~20% depending on the spectral position of *ω*_1_ and *ω*_2_ pumps. Considering the qualitative nature of our theoretical model, we have neglected the variation in Δ*N*_*e*_ to simplify calculations. More detailed analysis of the dependence of *χ*^(2)^(*ω*_1_, *ω*_2_) on pump frequencies and threshold gain should also include electron transport and the resultant non-uniform electric field distribution (band bending) across the active region. Such analysis can be performed using Monte-Carlo simulations^27^,^28^ in future work.

**Figure 6:**
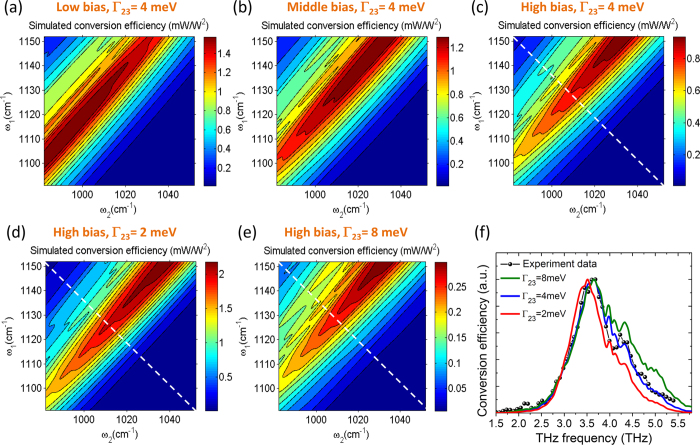
Calculated mid-IR-to-THz conversion efficiency for the tested devices as a function of mid-IR pump frequencies *ω*_1_ and *ω*_2_ assuming Γ_23_ = 4 meV near threshold (**a**), in the middle of dynamic range (**b**) and near the rollover point (**c–e**) Calculated mid-IR-to-THz conversion efficiency for tested devices as a function of mid-IR pump frequencies close to the rollover point and assuming Γ_23_ = 2 meV (**d**) and Γ_23_ = 8 meV (**e,f**) Cross section data comparison between theory and experiment. Black circles are experimental data taken along white dashed line in Fig. 4(f); red, blue and green lines are the theoretical values taken along white dashed line in panels (**d,c,e**). Theoretical data with the linewidth factor Γ_23_ = 4 meV provides the best match to the experimental data.

To simulate the mid-IR-to-THz conversion efficiency for different values of *ω*_1_ and *ω*_2_ at the three different bias points, we used a slab-waveguide approximation that treats the Cherenkov THz emission as a leaky waveguide mode. The procedure is described in detail in ref. 29 for Cherenkov second harmonic generation and is applied to THz DFG-QCL modeling in ref. 13. The results are shown in Fig. 6(a–c) assuming Γ_23_ = 4 meV. Theoretical dependence of conversion efficiency on *ω*_1_ and *ω*_2_ is in good qualitative agreement with the experimental measurements in Fig. 4(d–f), although experimental values of conversion efficiencies are approximately 3 times lower than expected. As discussed in Methods, THz power measurements were not corrected for collection efficiency owing to challenges in operating the THz power measurement setup (mid-IR powers were corrected for collection efficiency). Relatively low collection efficiency of THz radiation may explain the discrepancy between theory and experiment. Limitations of the slab-waveguide model and uncertainties in materials parameters may also contribute to the discrepancy between theory and experiment. Both theory and experiment show conversion efficiency for DFG at 3.57 THz drops by a factor of 1.5 as the bias field increases. This happens because the values of transition energies and transition dipole moments in Eq. (2) vary with the bias field, which leads to changes in the value of *χ*^(2)^ and the mid-IR-to-THz conversion efficiency. Also, at low-to-moderate bias fields, both experimental data and theory show nearly constant conversion efficiency for different combinations of mid-IR pump frequencies as long as the THz difference-frequency stays the same. At higher bias, both experimental data and theory shows that THz DFG conversion efficiency is maximized at specific combinations of *ω*_1_ and *ω*_2_, around 1130–1150 cm^−1^ and 1010–1030 cm^−1^, respectively. Given the simplicity of our model, the agreement is remarkable. The experimental and theoretical results demonstrate that careful selection of the spectral position of mid-IR pumps is critically important for optimization of THz DFG-QCL performance.

Finally, we used our 2D DFG spectroscopic analysis to deduce, for the first time, the approximate value of the intersubband transition linewidth Γ_23_ between lower laser states of the bound-to-continuum QCL active region. We note that it is virtually impossible to measure Γ_23_ by linear absorption spectroscopy since the electron population difference between these lower laser states is very small. In Fig. 6(c–e), we show simulated conversion efficiency at bias fields near the rollover point as a function of *ω*_1_ and *ω*_2_ assuming Γ_23_ ≈ 4 meV, 2 meV, and 8 meV for both QCL stacks, respectively. The best agreement between simulations and experimental measurements shown in Fig. 4(f) is achieved for Γ_23_ ≈ 4 meV. To clarify this point further, we compare the experimental and theoretical data points taken along the white dashed lines in Fig. 4(f) and Fig. 6(c–e). The results are shown in Fig. 6(f). The theoretical curve with Γ_23_ = 4 meV clearly provides the best fit with the experimental data. In particular, our theoretical model predicts a factor of 11, 6, and 3 drop in mid-IR-to-THz conversion efficiency at 5 THz compared to 3.6 THz for Γ_23_ = 2 meV, 4 meV, and 8 meV, respectively. The drop in mid-IR-to-THz conversion efficiency measured experimentally is approximately 6.5. A value of Γ_23_ = 4 meV is in a reasonable agreement with the 2Γ = 2.8 meV electroluminescence linewidth measured at 10 K in a lattice matched InGaAs/AlInAs/InP 3.6 THz QCL^30^ and a linewidth of 2Γ = 2.5 meV measured at ~10 K in a passive GaAs/Al_0.33_Ga_0.67_As quantum well structure at 4.7 THz^31^, considering the expected linewidth increase at room temperature.

In summary, we have performed a comprehensive investigation of the 2D spectroscopic dependence of THz DFG in QCLs with Cherenkov phase-matching on the positions of mid-IR pump frequencies. Our results demonstrate that mid-IR-to-THz conversion efficiency in Cherenkov THz DFG-QCLs may vary by a factor of 2 for the same THz DFG frequency, depending on the spectral position of the mid-IR pumps. We have also experimentally deduced the linewidth of THz transitions between the lower laser states, which is critical for the accurate modeling of *χ*^(2)^ and understanding the optical loss in the lower laser level structure of mid-IR QCLs. The 2D spectroscopic technique presented here is expected to play a crucial role in the analysis and future optimization in the performance of THz DFG-QCLs.

## Methods

### Device growth and fabrication

The laser structure was grown by molecular beam epitaxy (MBE) on a 350-μm-thick semi-insulating InP substrate. The growth commenced with a 200-nm-thick InGaAs current injection layer (Si,7 × 10^17^ cm^−3^), followed by a 3-μm-thick InP lower cladding (Si,1.5 × 10^16^ cm^−3^). The active region made with an InGaAs/InAlAs heterostructure lattice-matched to InP was grown next. It comprised of 33 repetitions of the bandstructure shown in Fig. 1(a) designed to have a gain peak at λ = 8.5 μm followed by 33 repetitions of the bandstructure shown in Fig. 1(b) that was designed to have a gain peak at λ = 9.5 μm. The layer sequence of the λ = 9.5 μm design, is **39**/22/**8**/60/**9**/59/**10**/52/**13**/43/**14**/38/**15**/36/**16**/34/**19**/33/**23**/32/**25**/32/**29**/31, and for the λ = 8.5 μm design is **43**/18/**7**/55/**9**/53/**11**/48/**14**/37/**15**/35/**16**/33/**18**/31/**20**/29/**24**/29/**26**/27/**30**/27, where the layer thicknesses are in angstroms, bold numbers indicate barriers, and underlined numbers indicate regions doped with Si to n = 2 × 10^17^ cm^−3^. The top cladding layer consisted of 3-μm-thick InP (Si, 1.5 × 10^16^ cm^−3^), followed by a 100-nm-thick InP layer (Si, 3 × 10^18^ cm^−3^) and terminated with a 10-nm-thick InGaAs contact layer (Si, 2 × 10^19^ cm^−3^). Samples were processed into 22-μm-wide dry-etched ridge waveguides via inductive plasma etching. The sidewalls of the ridges were insulated with a 400-nm-thick layer of silicon nitride, followed by Ti/Au (15 nm/800 nm) contact metallization. Given the insulating nature of the substrate, a lateral contact current injection scheme was employed. The wafer was cleaved into laser bars approximately 1-2 mm long. The 350-μm-thick InP substrate associated with the THz exit facet of the device was mechanically polished at a 30 degree angle for the THz Cherenkov wave out-coupling. Special care was taken to ensure that the laser waveguide facets are unaffected by polishing. Devices were then Indium soldered epi-side up onto copper holders and wire bonded.

### External cavity setup

The dual-grating experimental setup is shown in Fig. 2(a). The back facets of the Cherenkov THz DFG-QCL chips are coated with a two-layer anti-reflection (AR) coating using YF_3_ and ZnSe designed to have a minimum reflection around the middle of the gain bandwidth of the device^16^. An AR-coated aspheric lens with a focal length of 1.87 mm and a numerical aperture of 0.85 is used to collimate the mid-IR emission from the back facet of the laser. The collimated mid-IR beam is then split by a 50/50 ZnSe beam splitter and directed to two diffraction gratings (gold coated, 150 grooves mm^−1^) placed on two computer-controlled rotation stages. The gratings are used to provide frequency-tunable feedback for two mid-IR pump frequencies (*ω*_1_ and *ω*_2_) independently.

### Power and spectral measurements

Power measurements are done in a N_2_-purged environment using two parabolic mirrors: one parabolic mirror with a two inch focal length is used to collect and collimate light from the device and the other parabolic mirror with a six inch focal length is used to refocus the collimated beam onto a thermopile detector for mid-IR power measurements or a calibrated silicon bolometer for THz power measurements. Optical filters are used to spectrally separate the two mid-IR pumps. Mid-IR power is corrected for 70% collection efficiency of the setup. THz power measurements are not corrected for collection efficiency. Mid-IR and THz emission spectra of devices are measured with Nicolet iS50R Fourier-transform infrared spectrometer (FTIR) equipped with a deuterated L-alanine doped triglycine sulphate detector (DTGS) for mid-IR measurements and a helium-cooled silicon bolometer for terahertz measurements at a spectral resolution of 0.25 cm^−1^.

## Additional Information

**How to cite this article**: Jiang, Y. *et al.* Spectroscopic Study of Terahertz Generation in Mid-Infrared Quantum Cascade Lasers. *Sci. Rep.* **6**, 21169; doi: 10.1038/srep21169 (2016).
